# Supplementary material for: Green Extraction of Six Phenolic Compounds from Rattan (Calamoideae faberii) with Deep Eutectic Solvent by Homogenate-Assisted Vacuum-Cavitation Method
Source: Molecules. 2018 Dec 29;24(1):113. doi: 10.3390/molecules24010113 (PMC6337183; doi:10.3390/molecules24010113)
Supplement: Supplementary file 1 [file molecules-24-00113-s001.pdf]

**Supplementary Material**

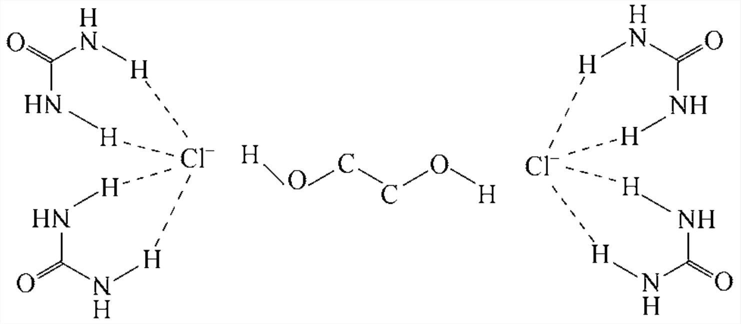


**Figure 1.** Formation diagram of hydrogen bonds between ChCl and EG.

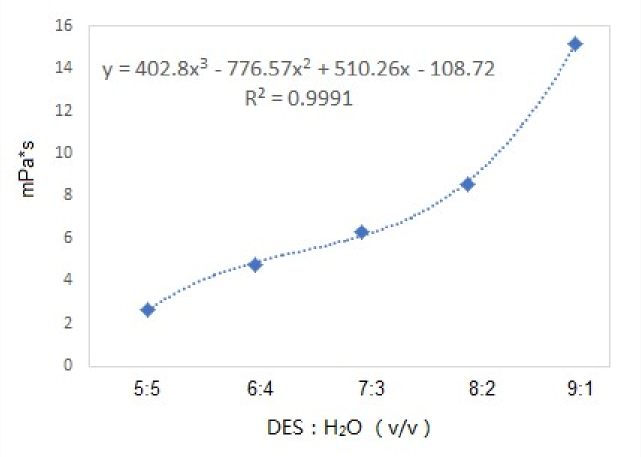


**Figure S2.** Effect of water content on the viscosity of DES solution

**Table S1.** Parameters of homogenate-assisted and vacuum-cavitation extraction

| No. | Operation | Solid-Liquid Ratio (g/mL) | Duration Time (min) | Solvent | Total Phenolic Content (mg/g) |
|---|---|---|---|---|---|
| 1-1 | homogenate | 1:15 | 2.0 | DES | 8.75 ± 0.16 |
| 1-2 | homogenate | 1:15 | 2.0 | Pure water | 1.97 ± 0.04 |
| 1-3 | homogenate | 1:15 | 2.0 | 80% methanol | 6.44 ± 0.11 |
| 2-1 | vacuum-cavitation | 1:15 | 25.0 | DES | 90.33 ± 1.89 |
| 2-2 | vacuum-cavitation | 1:15 | 25.0 | Pure water | 21.44 ± 0.44 |
| 2-3 | vacuum-cavitation | 1:15 | 25.0 | 80% methanol | 74.55 ± 1.23 |
| 3-1 | ultrasonic-assisted | 1:15 | 25.0 | DES | 84.32 ± 1.98 |
| 3-2 | ultrasonic-assisted | 1:15 | 25.0 | Pure water | 17.25 ± 0.64 |
| 3-3 | ultrasonic-assisted | 1:15 | 25.0 | 80% methanol | 64.83 ± 1.14 |
